# Disparities in State-Specific Adult Fruit and Vegetable Consumption — United States, 2015

**DOI:** 10.15585/mmwr.mm6645a1

**Published:** 2017-11-17

**Authors:** Seung Hee Lee-Kwan, Latetia V. Moore, Heidi M. Blanck, Diane M. Harris, Deb Galuska

**Affiliations:** 1 Division of Nutrition, Physical Activity, and Obesity, National Center for Chronic Disease Prevention and Health Promotion, CDC.

The *2015*–*2020 Dietary Guidelines for Americans* recommend that Americans consume more fruits and vegetables as part of an overall dietary pattern to reduce the risk for diet-related chronic diseases such as cardiovascular disease, type 2 diabetes, some cancers, and obesity (*1*). Adults should consume 1.5–2.0 cup equivalents of fruits and 2.0–3.0 cups of vegetables per day.* Overall, few adults in each state met intake recommendations according to 2013 Behavioral Risk Factor Surveillance System (BRFSS) data; however, sociodemographic characteristics known to be associated with fruit and vegetable consumption were not examined (*2*). CDC used data from the 2015 BRFSS to update the 2013 report and to estimate the percentage of each state’s population meeting intake recommendations by age, sex, race/ethnicity, and income-to-poverty ratio (IPR) for the 50 states and District of Columbia (DC). Overall, 12.2% of adults met fruit recommendations ranging from 7.3% in West Virginia to 15.5% in DC, and 9.3% met vegetable recommendations, ranging from 5.8% in West Virginia to 12.0% in Alaska. Intake was low across all socioeconomic groups. Overall, the prevalence of meeting the fruit intake recommendation was highest among women (15.1%), adults aged 31–50 years (13.8%), and Hispanics (15.7%); the prevalence of meeting the vegetable intake recommendation was highest among women (10.9%), adults aged ≥51 years (10.9%), and persons in the highest income group (11.4%). Evidence-based strategies that address barriers to fruit and vegetable consumption such as cost or limited availability could improve consumption and help prevent diet-related chronic disease.

BRFSS conducts an annual, state-based, random-digit–dialed landline and cellular telephone household survey of noninstitutionalized, civilian U.S. adults aged ≥18 years to collect data on health and health risk behaviors related to chronic disease. BRFSS uses a complex multistage cluster sampling design and weights by iterative proportional fitting to adjust for nonresponse, noncoverage, and selection bias (*3*). In 2015, BRFSS asked six questions to assess how many times per day, week, or month the participants consumed 1) 100% fruit juice, 2) whole fruit, 3) dried beans, 4) dark green vegetables, 5) orange vegetables, and 6) other vegetables, during the previous month. Daily frequency of intake was calculated by dividing reported intake by 7 for intake reported by week, and by 30 for intake reported by month. To estimate the percentage of each state’s population meeting fruit and vegetable intake recommendations by demographic characteristics, previously developed scoring algorithms derived from the National Health and Nutrition Examination Survey (NHANES) were used to predict whether a respondent met fruit and vegetable recommendations for their age and sex based on the number of times per day they reported consuming fruits and vegetables separately, accounting for race/ethnicity and IPR (poverty defined according to federal poverty guidelines^†^), updated with 2015 Poverty Guidelines for the 48 contiguous states and DC. IPR was calculated following the previous study methods (*4*) using the midpoint of reported household income for persons who reported their household income; household size was assumed to equal one for participants who did not report the number of persons residing in the household. Individual predicted probabilities of meeting recommendations were averaged to obtain sociodemographic-specific estimates. Intake recommendations were based on the *2015–2020 Dietary Guidelines for Americans* (*1*) and used the age- and sex-specific recommendations for adults who engage in <30 minutes of moderate physical activity daily. BRFSS respondents’ race/ethnicity (Hispanic, non-Hispanic black [black], non-Hispanic white [white], and all others) and IPR (<1.25, 1.25%–3.49%, and >3.49) were defined consistent with definitions in previous analyses (*5*). Estimates for the racial/ethnic group “other” are not presented because of the small sample sizes and difficulties in providing meaningful interpretation, but are included in overall estimates and those by age, sex, and IPR. Among 441,456 respondents, 122,041 (28%) were excluded, including 5,074 who did not reside in the 50 states or DC (because the scoring algorithm is derived from NHANES, which excluded territories), 58,949 who did not answer all six questions on fruit and vegetable intake, 127 who had implausible values of reported intake of fruit more than 16 times per day or vegetables more than 23 times per day (*4*), and 55,891 who had missing values for income, resulting in a final analytic sample of 319,415. The median response rate for 2015 BRFSS^§^ was 47.2% for the 50 states and DC (range = 33.9%–61.1%). T-tests were used to compare differences by demographic groups. Statistical analyses were performed to account for the complex survey design and nonresponse. Balanced repeated replication technique, replicate weights, and Taylor linearization were used to calculate standard errors and confidence intervals, consistent with the previous study (*2*).

In 2015, the median frequency of reported intake among all respondents was one time per day for fruit and 1.7 times per day for vegetables (Table 1). Among all respondents, 12.2% met fruit intake recommendations, ranging from 7.3% in West Virginia to 15.5% in DC, and 9.3% met vegetable intake recommendations, ranging from 5.8% in West Virginia to 12.0% in Alaska (Table 1). Overall in 2015, the percentage of adults meeting fruit and vegetable recommendations varied by selected characteristics (Table 2) (Table 3). A higher proportion of women met both fruit and vegetable recommendations (15.1% and 10.9%, respectively) than did men (9.2% and 7.6%, respectively), and a higher proportion of women met recommendations in most states. By age group, young adults aged 18–30 years accounted for the lowest proportion of persons meeting recommendations for fruit and vegetable intake (9.2% and 6.7%, respectively); this proportion was significantly different from the referent group of adults aged ≥51 years, 12.4% and 10.9% of whom met intake recommendations for fruit and vegetables, respectively. Findings varied by state; in 41 states, a significantly lower percentage of young adults met recommendations for vegetable intake than did older adults, whereas this pattern was only observed for fruit intake in 18 states. A significantly higher proportion of Hispanics and blacks met recommendations for fruit intake than did whites; however, these differences were only significant in 10 states (Table 2). There were no significant differences in meeting recommendations for vegetable intake by race/ethnicity. In general, by state, lower percentages of blacks met recommendation for vegetable intake than did whites and Hispanics. Overall, a significantly higher percentage of persons with IPR >3.49 met recommendations for vegetable intake than did those with IPR ≤3.49, although no significant differences for meeting recommendations for fruit intake by IPR were observed. By state, a higher percentage of persons living in households with incomes in the highest category (IPR >3.49) met the recommendation for vegetable intake than did persons living below or close to the poverty level (IPR <1.25); these differences were significant in four states for fruits and 35 states for vegetables.

**TABLE 1:** Median frequencies and percentages of adults meeting federal fruit and vegetable intake recommendations per day, by state — Behavioral Risk Factor Surveillance System, United States and District of Columbia, 2015

Jurisdiction	Sample size	Median daily intake frequency		% of respondents (95% CI) meeting recommendations	
		Fruit	Vegetable	Fruit intake	Vegetable intake
**Overall**	**319,415**	**1.0**	**1.7**	**12.2 (11.2−13.3)**	**9.3 (6.1−12.5)**
Alabama	5,630	1.0	1.5	8.8 (7.3−10.2)	6.1 (2.8−9.4)
Alaska	2,944	1.0	1.8	12.9 (10.5−15.2)	12.0 (6.6−17.4)
Arizona	5,490	1.0	1.7	13.2 (11.4−15.1)	10.5 (6.0−14.9)
Arkansas	3,631	0.9	1.5	9.5 (7.5−11.5)	7.8 (3.7−12.0)
California	9,443	1.1	1.8	13.6 (12.0−15.2)	11.2 (7.0−15.4)
Colorado	9,530	1.1	1.8	13.5 (11.7−15.3)	11.6 (7.6−15.6)
Connecticut	8,491	1.1	1.7	13.5 (11.8−15.1)	10.4 (6.8−13.9)
Delaware	2,859	1.0	1.7	12.5 (10.4−14.5)	8.6 (5.0−12.1)
District of Columbia	2,814	1.0	1.9	15.5 (12.5−18.6)	9.7 (4.7−14.8)
Florida	7,152	1.0	1.7	14.0 (12.2−15.8)	10.3 (6.7−13.8)
Georgia	3,342	1.0	1.6	12.0 (10.1−13.9)	8.5 (4.7−12.4)
Hawaii	5,900	1.0	1.7	12.4 (10.6−14.1)	11.5 (7.4−15.6)
Idaho	4,616	1.0	1.8	11.8 (9.9−13.6)	10.8 (6.9−14.6)
Illinois	4,585	1.0	1.6	14.0 (12.1−15.9)	9.7 (5.7−13.7)
Indiana	4,596	1.0	1.5	11.5 (9.6−13.4)	8.6 (4.5−12.6)
Iowa	4,706	1.0	1.5	10.7 (9.0−12.4)	7.0 (3.5−10.6)
Kansas	16,780	1.0	1.6	10.0 (8.8−11.2)	8.1 (4.8−11.5)
Kentucky	5,074	1.0	1.5	8.0 (6.6−9.5)	6.3 (2.4−10.2)
Louisiana	3,293	0.9	1.4	11.2 (9.3−13.0)	8.3 (4.6−12.1)
Maine	7,447	1.1	1.7	14.1 (12.2−16.0)	10.7 (7.3−14.1)
Maryland	8,800	1.1	1.7	14.0 (11.9−16.0)	9.0 (5.3−12.7)
Massachusetts	6,198	1.1	1.7	14.0 (12.2−15.7)	11.1 (7.3−14.8)
Michigan	6,835	1.0	1.6	11.9 (10.3−13.5)	7.7 (4.3−11.1)
Minnesota	12,789	1.0	1.6	11.6 (10.2−13.1)	8.1 (4.8−11.4)
Mississippi	4,416	0.9	1.4	8.7 (7.1−10.4)	6.2 (2.7−9.7)
Missouri	5,326	1.0	1.6	10.8 (9.1−12.4)	7.7 (3.9−11.5)
Montana	4,528	1.0	1.7	10.7 (8.8−12.5)	8.3 (4.5−12.1)
Nebraska	13,771	1.0	1.6	11.4 (9.9−12.9)	7.9 (4.2−11.6)
Nevada	2,133	1.0	1.8	13.1 (10.5−15.6)	11.5 (7.0−16.0)
New Hampshire	5,005	1.1	1.8	14.3 (12.2−16.4)	10.8 (7.2−14.4)
New Jersey	8,207	1.0	1.6	12.1 (10.5−13.8)	8.3 (4.7−11.8)
New Mexico	5,166	1.0	1.7	12.3 (10.5−14.0)	10.3 (6.1−14.6)
New York	9,145	1.0	1.7	14.0 (12.4−15.6)	9.6 (5.9−13.2)
North Carolina	4,737	1.0	1.7	10.4 (8.9−12.0)	8.1 (4.6−11.6)
North Dakota	3,911	1.0	1.5	11.9 (10.0−13.7)	7.9 (3.7−12.0)
Ohio	8,495	1.0	1.6	10.6 (9.0−12.2)	6.9 (3.3−10.5)
Oklahoma	5,177	1.0	1.6	8.0 (6.6−9.5)	6.1 (2.6−9.6)
Oregon	3,848	1.0	1.9	13.4 (11.4−15.4)	11.9 (8.0−15.8)
Pennsylvania	4,287	1.0	1.6	11.7 (9.9−13.5)	8.4 (4.6−12.1)
Rhode Island	4,249	1.0	1.6	13.7 (11.7−15.7)	9.7 (6.0−13.5)
South Carolina	8,485	1.0	1.6	10.1 (8.7−11.6)	8.1 (4.8−11.5)
South Dakota	5,511	1.0	1.5	8.8 (7.2−10.5)	5.9 (2.6−9.3)
Tennessee	4,115	1.0	1.6	11.1 (9.2−13.1)	9.6 (5.6−13.6)
Texas	10,433	1.0	1.7	12.1 (10.4−13.8)	10.9 (6.6−15.1)
Utah	8,737	1.0	1.7	12.5 (10.9−14.1)	9.4 (5.1−13.7)
Vermont	4,877	1.1	1.8	12.7 (10.9−14.5)	11.1 (7.5−14.6)
Virginia	6,593	1.0	1.6	10.9 (9.3−12.5)	7.6 (3.9−11.4)
Washington	12,247	1.0	1.8	12.6 (11.1−14.2)	10.9 (7.1−14.8)
West Virginia	4,200	1.0	1.5	7.3 (6.0−8.7)	5.8 (2.7−8.9)
Wisconsin	4,894	1.0	1.6	11.7 (10.0−13.5)	7.8 (4.0−11.5)
Wyoming	3,977	1.0	1.7	12.1 (9.9−14.2)	9.1 (4.8−13.5)

**Abbreviation:** CI = confidence interval.

**TABLE 2:** State-specific percentages of respondents meeting federal fruit intake recommendations* by sex, age, race/ethnicity, and income-to-poverty ratio (IPR) — Behavioral Risk Factor Surveillance System, United States, 2015

Jurisdiction	Sex		Age group (yrs)			Race/Ethnicity^†^			IPR		
	Male	Female (Ref)	18–30	31–50	≥51 (Ref)	Black	Hispanic	White (Ref)	<1.25	1.25–3.49	>3.49 (Ref)
	% (95% CI)	% (95% CI)	% (95% CI)	% (95% CI)	% (95% CI)	% (95% CI)	% (95% CI)	% (95% CI)	% (95% CI)	% (95% CI)	% (95% CI)
**National**	**9.2 (5.8−12.7)**	**15.1^¶^ (11.8−18.7)**	**9.2^¶^ (7.1−11.4)**	**13.8 (11.9−15.9)**	**12.4 (11.0−13.8)**	**14.3^¶^ (12.5−16.2)**	**15.7^¶^ (13.5−17.8)**	**11.2 (10.1−12.3)**	**11.9 (10.6−13.6)**	**11.3 (9.9−12.8)**	**13.0 (11.6−14.3)**
Alabama	7.5 (4.0−11.0)	10.0 (6.4−13.5)	8.4 (4.8−12.1)	9.6 (6.9−12.3)	8.3 (6.6−10.0)	11.6^¶^ (8.6−14.6)	11.1 (4.6−17.5)	7.6 (6.1−9.1)	9.1 (6.4−11.9)	7.7 (5.7−9.7)	9.5 (7.5−11.4)
Alaska	10.0 (4.4−15.7)	16.1^¶^ (10.4−21.8)	9.5 (4.1−14.9)	15.4 (11.0−19.9)	12.8 (9.9−15.7)	—^§^	14.6 (6.5−22.6)	12.9 (10.2−15.5)	11.1 (6.1−16.1)	11.3 (8.0−14.6)	14.4 (11.1−17.7)
Arizona	10.4 (5.7−15.0)	16.2^¶^ (11.5−20.8)	10.4 (5.9−15.0)	15.9 (12.4−19.4)	12.5 (10.4−14.7)	19.2 (9.3−29.1)	15.9^¶^ (11.9−19.8)	12.1 (10.1−14.0)	13.6 (10.1−17.1)	11.0^¶^ (8.4−13.6)	15.0 (12.4−17.6)
Arkansas	7.3 (2.9−11.7)	11.7^¶^ (7.3−16.1)	9.6 (3.7−15.6)	10.4 (6.9−13.8)	8.7 (6.8−10.7)	11.5 (5.4−17.5)	16.4 (7.7−25.1)	8.6 (6.6−10.6)	10.0 (6.5−13.5)	9.1 (6.1−12.0)	9.6 (6.8−12.4)
California	10.4 (6.0−14.8)	16.8^¶^ (12.4−21.2)	9.3^¶^ (6.2−12.5)	16.0 (13.1−18.9)	13.8 (11.5−16.0)	14.1 (9.6−18.5)	15.9 (13.1−18.7)	12.4 (10.6−14.1)	13.9 (11.2−16.6)	13.0 (10.5−15.5)	13.8 (11.8−15.8)
Colorado	10.2 (6.0−14.4)	17.0^¶^ (12.8−21.1)	9.4 (5.4−13.4)	16.3 (12.7−19.8)	13.2 (10.9−15.2)	18.7 (10.2−27.1)	16.5 (12.4−20.6)	12.5 (10.6−14.4)	12.2 (8.9−15.5)	12.3 (9.6−15.1)	14.6 (12.3−17.0)
Connecticut	9.7 (5.9−13.4)	17.2^¶^ (13.4−20.9)	8.6^¶^ (4.7−12.5)	14.9 (11.8−18.1)	14.4 (12.3−16.5)	16.1 (11.8−20.4)	14.7 (10.6−18.8)	13.1 (11.3−14.9)	11.7 (8.9−14.4)	13.0 (10.4−15.5)	14.2 (12.1−16.3)
Delaware	8.9 (5.2−12.7)	15.9^¶^ (12.1−19.6)	7.3^¶^ (2.9−11.7)	14.3 (10.3−18.3)	13.0 (10.5−15.6)	16.9 (12.0−21.8)	14.4 (8.0−20.7)	11.1 (8.9−13.3)	9.8 (6.4−13.3)	11.0 (8.1−14.0)	14.1 (11.3−17.0)
District of Columbia	13.4 (8.1−18.8)	17.5 (12.2−22.8)	9.1 (2.8−15.5)	21.2^¶^ (15.4−26.9)	13.8 (11.0−16.5)	15.9 (11.4−20.4)	25.9 (13.1−38.7)	13.5 (9.7−17.3)	19.4 (12.3−26.5)	14.7 (8.3−21.2)	14.8 (11.2−18.3)
Florida	11.2 (7.4−14.9)	16.8^¶^ (13.0−20.5)	13.9 (8.6−19.2)	15.9 (12.6−19.2)	12.9 (10.9−14.8)	16.9 (12.4−21.4)	16.6 (12.5−20.7)	12.2 (10.3−14.1)	13.2 (10.2−16.1)	13.6 (10.7−16.5)	14.7 (12.4−17.1)
Georgia	8.9 (4.8−12.9)	14.8^¶^ (10.7−18.9)	9.1 (4.7−13.5)	14.4 (10.7−18.2)	11.2 (9.0−13.4)	12.5 (9.3−15.7)	18.1 (9.8−26.3)	10.8 (8.7−13.0)	11.2 (7.6−14.8)	11.8 (8.8−14.8)	12.5 (10.0−15.1)
Hawaii	9.8 (5.5−14.1)	14.9^¶^ (10.7−19.2)	9.9 (5.4−14.4)	12.5 (9.4−15.7)	13.3 (11.2−15.5)	—^§^	15.4 (10.2−20.5)	14.8 (11.8−17.8)	12.7 (9.4−16.1)	11.7 (9.0−14.5)	12.6 (10.4−14.8)
Idaho	8.4 (4.4−12.4)	15.1^¶^ (11.1−19.1)	8.3 (4.3−12.3)	13.2 (9.5−16.9)	12.1 (9.9−14.3)	—^§^	12.9 (7.8−18.0)	11.7 (9.7−13.6)	11.3 (7.7−15.0)	10.9 (8.3−13.5)	12.8 (10.4−15.2)
Illinois	11.1 (6.8−15.3)	16.9^¶^ (12.6−21.1)	10.7 (6.4−15.0)	15.8 (12.1−19.5)	14.4 (12.1−16.7)	14.7 (10.2−19.2)	19.8^¶^ (14.2−25.3)	12.8 (10.8−14.7)	13.3 (9.6−17.0)	13.1 (10.3−16.0)	14.9 (12.4−17.3)
Indiana	8.8 (4.5−13.1)	14.1^¶^ (9.8−18.4)	9.9 (5.2−14.7)	13.2 (9.4−17.0)	11.0 (8.9−13.1)	13.3 (7.6−19.0)	18.2 (9.5−26.9)	10.9 (8.9−12.9)	12.0 (7.6−16.4)	9.8 (7.3−12.3)	12.7 (10.0−15.4)
Iowa	7.0 (3.3−10.8)	14.3^¶^ (10.6−18.1)	7.3^¶^ (3.2−11.3)	11.1 (7.8−14.4)	11.9 (9.8−14.0)	7.9 (2.6−13.2)	15.6 (6.9−24.4)	10.4 (8.7−12.1)	9.4 (6.2−12.6)	10.0 (7.4−12.7)	11.4 (9.3−13.6)
Kansas	6.7 (3.1−10.3)	13.2^¶^ (9.6−16.8)	6.4^¶^ (4.1−8.8)	11.1 (8.7−13.4)	10.8 (9.2−12.4)	12.0 (8.2−15.7)	12.6 (9.5−15.6)	9.6 (8.3−10.9)	8.7 (6.7−10.7)	9.2 (7.5−10.9)	11.0 (9.5−12.6)
Kentucky	5.7 (1.5−9.9)	10.5^¶^ (6.4−14.7)	5.5 (2.3−8.7)	8.2 (5.5−10.8)	9.2 (7.1−11.3)	8.5 (4.7−12.4)	5.5 (1.7−9.3)	8.1 (6.5−9.7)	6.9 (4.7−9.1)	7.2 (4.8−9.5)	9.3 (7.3−11.4)
Louisiana	9.9 (5.9−13.9)	12.4^¶^ (8.4−16.4)	8.7 (4.7−12.7)	13.6 (9.9−17.3)	10.4 (8.2−12.5)	13.7 (10.3−17.1)	14.5 (6.1−23.0)	10.0 (7.8−12.1)	11.4 (8.0−14.7)	10.7 (7.8−13.6)	11.5 (8.8−14.1)
Maine	9.7 (6.1−13.2)	18.4^¶^ (14.8−22.0)	11.5 (6.6−16.4)	14.7 (10.7−18.7)	14.7 (12.5−16.9)	—^§^	20.0 (7.6−32.3)	14.1 (12.2−16.1)	10.7^¶^ (7.6−13.7)	13.9 (11.0−16.8)	15.5 (13.1−18.0)
Maryland	10.9 (6.9−14.8)	16.8^¶^ (12.8−20.7)	10.6 (5.5−15.6)	15.2 (11.4−19.1)	14.5 (12.0−17.0)	18.0^¶^ (13.7−22.2)	13.6 (7.1−20.1)	12.2 (10.2−14.3)	14.4 (9.4−19.4)	13.8 (10.3−17.3)	14.0 (11.6−16.4)
Massachusetts	10.3 (6.4−14.3)	17.4^¶^ (13.4−21.3)	8.8^¶^ (5.4−12.1)	15.8 (12.4−19.2)	14.9 (12.6−17.3)	18.3 (12.3−24.3)	17.8 (12.9−22.6)	13.5 (11.6−15.4)	12.5 (9.3−15.6)	13.3 (10.5−16.1)	14.6 (12.4−16.8)
Michigan	8.3 (4.6−11.9)	15.5^¶^ (11.9−19.2)	9.0^¶^ (5.3−12.6)	12.8 (9.7−15.9)	12.5 (10.4−14.5)	14.8 (11.0−18.6)	16.1 (10.1−22.1)	11.4 (9.7−13.1)	11.7 (8.6−14.9)	10.4 (8.2−12.6)	13.1 (11.0−15.2)
Minnesota	7.7 (4.2−11.2)	15.6^¶^ (12.1−19.1)	7.8^¶^ (4.9−10.7)	12.4 (9.6−15.1)	12.8 (10.9−14.7)	13.1 (8.8−17.4)	14.7 (9.6−19.8)	11.6 (10.1−13.1)	9.5^¶^ (7.0−12.1)	10.1^¶^ (8.1−12.0)	13.0 (11.2−14.8)
Mississippi	7.1 (3.3−10.8)	10.3 (6.5−14.1)	9.5 (5.3−13.7)	9.5 (6.7−12.4)	7.7 (5.9−9.4)	10.0 (7.2−12.8)	—	7.8 (6.1−9.6)	8.5 (5.9−11.1)	7.8 (5.5−10.2)	10.1 (7.7−12.5)
Missouri	7.9 (3.9−12.0)	13.6^¶^ (9.6−17.7)	8.2 (4.3−12.1)	11.3 (8.1−14.5)	11.6 (9.5−13.6)	15.1 (10.4−19.9)	11.9 (3.7−20.2)	10.1 (8.4−11.8)	9.4 (6.3−12.5)	10.1 (7.7−12.4)	11.8 (9.6−14.1)
Montana	7.3 (3.3−11.3)	14.1^¶^ (10.1−18.1)	8.1 (3.6−12.5)	11.2 (7.6−14.8)	11.3 (9.1−13.5)	—^§^	14.3 (5.8−22.8)	10.5 (8.6−12.4)	9.5 (6.3−12.7)	10.2 (7.4−13.0)	11.5 (9.2−13.8)
Nebraska	8.4 (4.5−12.3)	14.4^¶^ (10.5−18.3)	8.1^¶^ (4.9−11.3)	12.0 (9.1−14.9)	12.5 (10.6−14.5)	17.4^¶^ (10.7−24.2)	17.4^¶^ (12.2−22.5)	10.5 (9.0−12.0)	10.7 (8.0−13.3)	11.2 (9.0−13.5)	11.7 (9.9−13.6)
Nevada	11.2 (6.6−15.9)	15.0^¶^ (10.4−19.7)	13.6 (6.3−20.9)	13.8 (9.4−18.1)	12.2 (9.1−15.2)	18.2 (8.4−28.0)	11.5 (7.1−15.9)	12.8 (9.8−15.9)	18.3 (11.2−25.3)	10.3 (7.1−13.6)	13.1 (9.7−16.5)
New Hampshire	10.8 (7.0−14.6)	17.8^¶^ (14.0−21.6)	11.5 (5.2−17.7)	14.9 (10.9−18.9)	14.9 (12.5−17.3)	—^§^	—^§^	14.6 (12.4−16.8)	13.2 (8.6−17.8)	11.6^¶^ (8.7−14.5)	16.1 (13.4−18.7)
New Jersey	9.5 (5.7−13.2)	14.7^¶^ (10.9−18.5)	8.4^¶^ (4.6−12.1)	12.8 (9.9−15.7)	13.2 (10.9−15.5)	15.1 (11.2−19.1)	12.3 (9.0−15.7)	11.3 (9.5−13.2)	10.8 (7.7−13.9)	12.0 (9.4−14.6)	12.6 (10.5−14.7)
New Mexico	8.8 (4.4−13.2)	15.6^¶^ (11.2−20.0)	8.0 (4.5−11.5)	15.6 (12.0−19.1)	11.8 (9.5−14.1)	25.8^¶^ (12.3−39.3)	12.8 (10.0−15.6)	10.4 (8.5−12.3)	13.2 (10.2−16.2)	10.2 (7.9−12.6)	13.4 (10.8−16.1)
New York	10.6 (6.7−14.4)	17.2^¶^ (13.3−21.1)	9.4^¶^ (6.2−12.6)	15.8 (12.7−18.9)	14.8 (12.7−16.8)	17.0 (13.1−21.0)	17.0 (13.4−20.6)	13.3 (11.5−15.0)	13.8 (11.0−16.5)	13.3 (11.0−15.7)	14.5 (12.4−16.6)
North Carolina	7.8 (4.1−11.5)	12.9^¶^ (9.2−16.7)	7.7 (4.4−11.0)	12.5 (9.6−15.4)	9.8 (7.9−11.8)	13.4^¶^ (10.1−16.8)	16.4^¶^ (11.2−21.5)	8.9 (7.3−10.5)	10.1 (7.3−12.8)	9.9 (7.5−12.3)	11.0 (9.0−12.9)
North Dakota	8.3 (3.8−12.7)	15.7^¶^ (11.3−20.1)	7.1^¶^ (3.2−10.9)	12.5 (8.9−16.2)	14.0 (11.6−16.4)	—^§^	—^§^	11.1 (9.3−12.9)	9.2 (5.7−12.8)	11.6 (8.5−14.6)	12.6 (10.3−14.8)
Ohio	7.5 (3.7−11.3)	13.7^¶^ (9.9−17.6)	9.1 (5.0−13.2)	10.8 (7.8−13.8)	11.1 (9.2−13.0)	13.1 (8.3−17.9)	20.5 (9.8−31.2)	9.8 (8.2−11.4)	10.8 (7.4−14.1)	9.2 (7.0−11.4)	11.6 (9.5−13.8)
Oklahoma	5.6 (1.9−9.3)	10.3^¶^ (6.6−14.0)	4.8^¶^ (1.8−7.8)	9.5 (6.5−12.4)	8.3 (6.6−10.0)	11.1 (5.9−16.2)	11.4 (6.4−16.3)	7.4 (5.9−8.9)	6.7 (4.1−9.3)	8.2 (6.0−10.4)	8.5 (6.7−10.3)
Oregon	9.6 (5.6−13.7)	17.0^¶^ (13.0−21.1)	8.6^¶^ (4.2−12.9)	14.6 (10.7−18.4)	14.5 (12.0−17.0)	—^§^	16.1 (9.4−22.8)	13.2 (11.1−15.2)	11.0 (7.1−15.0)	12.8 (9.9−15.8)	14.6 (12.1−17.1)
Pennsylvania	8.6 (4.6−12.6)	14.7^¶^ (10.8−18.7)	8.3 (4.4−12.3)	12.8 (9.2−16.4)	12.4 (10.2−14.6)	15.7 (10.8−20.6)	14.1 (6.6−21.6)	11.2 (9.3−13.0)	11.8 (7.1−16.5)	10.3 (7.9−12.7)	12.7 (10.5−14.9)
Rhode Island	10.4 (6.5−14.4)	16.8^¶^ (12.9−20.7)	11.8 (6.3−17.3)	13.5 (9.9−17.1)	14.6 (12.2−17.1)	14.5 (6.8−22.3)	17.5 (11.1−24.0)	12.8 (10.8−14.8)	14.8 (10.5−19.1)	11.9 (8.9−15.0)	14.5 (11.9−17.1)
South Carolina	7.6 (4.0−11.2)	12.5^¶^ (8.9−16.1)	7.9 (4.7−11.1)	12.2 (9.4−14.9)	9.6 (7.9−11.4)	11.4 (8.9−13.8)	24.4^¶^ (15.6−33.2)	8.7 (7.3−10.2)	10.9 (8.2−13.7)	9.8 (7.7−11.8)	10.1 (8.3−11.9)
South Dakota	5.9 (2.3−9.6)	11.9^¶^ (8.3−15.5)	5.9 (2.2−9.7)	9.9 (6.7−13.2)	9.2 (7.3−11.2)	—^§^	13.5 (2.0−24.9)	8.8 (7.1−10.5)	8.2 (4.6−11.8)	8.3 (6.0−10.7)	9.4 (7.4−11.5)
Tennessee	8.4 (4.2−12.6)	13.9^¶^ (9.7−18.1)	9.5 (4.8−14.1)	12.9 (9.1−16.8)	10.5 (8.4−12.5)	14.4 (9.0−19.7)	16.4 (4.8−28.0)	10.3 (8.4−12.3)	10.1 (6.5−13.8)	10.5 (7.7−13.3)	12.4 (9.7−15.0)
Texas	9.3 (4.9−13.8)	14.9^¶^ (10.4−19.3)	8.4 (4.8−12.1)	14.8 (11.7−17.9)	11.6 (9.4−13.8)	13.1 (8.7−17.5)	14.2^¶^ (11.1−17.3)	10.4 (8.5−12.2)	12.9 (9.8−16.0)	11.6 (9.0−14.3)	12.1 (9.8−14.3)
Utah	9.1 (4.5−13.7)	15.9^¶^ (11.3−20.5)	8.0^¶^ (5.0−11.0)	14.3 (11.3−17.4)	13.8 (11.6−16.0)	—^§^	15.7 (11.7−19.7)	12.2 (10.4−13.9)	10.8 (7.8−13.9)	11.7 (9.2−14.2)	13.5 (11.5−15.5)
Vermont	8.7 (5.0−12.4)	16.7^¶^ (13.0−20.4)	8.1^¶^ (4.0−12.3)	12.5 (9.1−15.9)	14.6 (12.3−17.0)	—^§^	—^§^	12.7 (10.9−14.6)	9.2^¶^ (6.6−11.9)	10.9^¶^ (8.4−13.5)	14.9 (12.5−17.3)
Virginia	8.5 (4.5−12.5)	13.2^¶^ (9.2−17.2)	9.7 (6.0−13.3)	11.6 (8.8−14.4)	10.9 (8.9−13.0)	13.0 (9.7−16.3)	14.2 (8.9−19.5)	9.8 (8.2−11.5)	10.3 (7.1−13.5)	10.1 (7.7−12.5)	11.5 (9.6−13.5)
Washington	9.2 (5.1−13.2)	16.1^¶^ (12.0−20.1)	8.9^¶^ (5.7−12.1)	13.9 (10.9−16.9)	13.4 (11.4−15.4)	17.1 (10.6−23.6)	16.2 (12.0−20.4)	12.0 (10.4−13.6)	13.9 (10.7−17.1)	11.6 (9.3−14.0)	12.9 (11.1−14.7)
West Virginia	5.2 (1.8−8.5)	9.5^¶^ (6.2−12.9)	6.1 (2.8−9.3)	7.4 (4.9−10.0)	7.8 (6.1−9.5)	9.4 (3.7−15.2)	—^§^	7.2 (5.8−8.6)	5.8^¶^ (3.8−7.9)	6.5^¶^ (4.7−8.3)	9.2 (7.2−11.2)
Wisconsin	8.0 (4.0−11.9)	15.6^¶^ (11.6−19.6)	8.2^¶^ (4.4−12.0)	12.4 (9.1−15.7)	12.9 (10.6−15.2)	17.9 (9.7−26.1)	15.6 (8.5−22.6)	11.2 (9.5−13.0)	12.2 (8.4−16.0)	10.4 (8.0−12.7)	12.7 (10.4−15.0)
Wyoming	9.0 (4.5−13.6)	15.3^¶^ (11.8−18.7)	10.7 (5.1−16.4)	13.9 (10.0−17.9)	11.4 (11.0−13.8)	—^§^	17.4 (9.2−25.6)	10.6 (8.7−12.6)	13.6 (7.3−19.9)	11.0 (7.8−14.2)	12.6 (10.0−15.1)

**Abbreviations:** CI = confidence interval; Ref = referent group.

* Weighted percentages are presented.

^†^ Blacks and whites are non-Hispanic; Hispanic persons could be of any race. Other racial/ethnic group not reported because of small sample sizes but included in overall estimates and estimates by other demographic characteristics.

^§^ Data where the sample sizes were <50 were considered unstable and were not reported.

^¶^ p<0.05 for t-test comparing differences by demographic groups to the referent group.

**TABLE 3:** State-specific percentages* of respondents meeting federal vegetable intake recommendations sex, age, race/ethnicity, and income-to-poverty ratio (IPR) — Behavioral Risk Factor Surveillance System, 2015

State	Sex (n = 319,415)		Age group (yrs, n = 319,415)			Race/Ethnicity^†^			IPR (n = 319,415)		
	Men	Women (Ref)	18–30	31–50	≥51 (Ref)	Black	Hispanic	White (Ref)	<1.25	1.25–3.49	>3.49 (Ref)
	% (95% CI)	% (95% CI)	% (95% CI)	% (95% CI)	% (95% CI)	% (95% CI)	% (95% CI)	% (95% CI)	% (95% CI)	% (95% CI)	% (95% CI)
**National**	**7.6 (5.6−9.6)**	**10.9^¶^ (9.0−13.0)**	**6.7^¶^ (4.7−8.7)**	**8.8^¶^ (6.9−10.9)**	**10.9 (8.9−12.9)**	**5.5 (0.0−11.1)**	**10.5 (5.0−16.1)**	**9.5 (3.9−15.1)**	**7.0^¶^ (3.9−10.4)**	**7.5^¶^ (4.3−10.8)**	**11.4 (8.2−14.7)**
Alabama	5.4 (3.3−7.5)	6.7 (4.6−8.8)	3.0^¶^ (0.7−5.3)	5.3^¶^ (3.0−7.7)	8.0 (5.7−10.3)	3.1 (0.0−11.3)	—^§^	7.0 (0.0−15.2)	3.4^¶^ (0.0−7.2)	4.9 (1.2−8.7)	8.4 (4.6−12.1)
Alaska	8.9 (4.2−13.5)	15.6^¶^ (10.9−20.2)	9.9 (6.1−13.7)	11.7 (7.9−15.5)	13.7 (9.8−17.5)	—^§^	13.2 (4.3−22.1)	12.9 (4.0−21.8)	11.7 (6.1−17.3)	9.4 (3.8−15.0)	13.9 (8.3−19.5)
Arizona	8.0 (4.7−11.2)	13.0^¶^ (9.8−16.3)	7.7^¶^ (4.8−10.5)	10.6 (7.7−13.4)	11.8 (9.0−14.7)	5.2 (0.0−14.9)	11.4 (1.7−21.1)	10.4 (0.7−20.1)	10.1 (5.8−14.4)	8.1^¶^ (3.8−12.3)	12.8 (8.5−17.0)
Arkansas	6.9 (4.2−9.6)	8.8 (6.1−11.5)	5.9^¶^ (3.1−8.8)	6.6^¶^ (3.8−9.5)	9.8 (6.9−12.6)	—^§^	9.9 (3.0−16.8)	8.4 (1.5−15.3)	6.3 (1.1−11.5)	6.6 (1.4−11.8)	10.2 (5.0−15.5)
California	9.3 (6.3−12.3)	13.0^¶^ (10.1−16.0)	7.5^¶^ (4.4−10.5)	11.2 (8.2−14.2)	13.3 (10.2−16.3)	6.6 (0.3−13.0)	10.6 (4.3−17.0)	11.8 (5.5−18.2)	9.3 (5.1−13.5)	9.1 (4.9−13.4)	13.1 (8.9−17.3)
Colorado	8.6 (5.3−11.8)	14.6^¶^ (11.4−17.9)	8.8^¶^ (5.9−11.8)	11.0 (8.0−13.9)	13.5 (10.5−16.4)	8.6 (0.2−17.1)	11.9 (3.5−20.3)	11.5 (3.1−20.0)	9.4 (5.3−13.4)	10.0 (6.0−14.1)	13.1 (9.1−17.2)
Connecticut	7.8 (4.7−10.8)	12.9^¶^ (9.8−15.9)	9.5 (6.9−12.1)	9.3 (6.7−11.9)	11.5 (8.9−14.1)	5.2 (0.0−13.8)	8.7 (0.2−17.3)	11.1 (2.6−19.7)	7.9^¶^ (4.0−11.8)	8.1^¶^ (4.2−12.0)	12.1 (8.2−16.0)
Delaware	6.4 (3.0−9.7)	10.6^¶^ (7.3−14.0)	5.7^¶^ (2.6−8.7)	7.5 (4.5−10.6)	10.4 (7.4−13.4)	6.3 (0.0−16.3)	5.6 (0.0−15.6)	9.1 (0.0−19.1)	4.6^¶^ (0.6−8.5)	6.2^¶^ (2.2−10.1)	11.1 (7.1−15.1)
District of Columbia	8.4 (3.9−12.9)	10.9 (6.4−15.4)	5.0^¶^ (1.6−8.3)	10.9 (7.5−14.3)	12.0 (8.6−15.3)	6.3 (0.0−17.0)	14.2 (3.4−24.9)	11.6 (0.8−22.3)	6.2 (0.3−12.1)	7.1 (1.2−13.0)	11.5 (5.6−17.5)
Florida	8.2 (5.4−11.1)	12.2^¶^ (9.4−15.1)	9.5 (7.0−12.0)	9.3 (6.8−11.8)	11.2 (8.7−13.7)	6.7 (0.0−16.1)	8.7 (0.0−18.1)	11.1 (1.7−20.5)	7.9^¶^ (3.7−12.1)	7.3^¶^ (3.2−11.5)	13.6 (9.4−17.8)
Georgia	6.4 (3.5−9.3)	10.5^¶^ (7.6−13.4)	6.8^¶^ (4.1−9.5)	8.1 (5.4−10.8)	9.8 (7.1−12.4)	5.4 (0.0−15.2)	7.1 (0.0−16.9)	10.1 (0.3−19.9)	4.5^¶^ (0.1−8.9)	7.6 (3.3−12.0)	11.2 (6.8−15.6)
Hawaii	9.7 (6.4−12.9)	13.5^¶^ (10.2−16.7)	7.9^¶^ (4.7−11.0)	10.2^¶^ (7.0−13.4)	14.2 (11.0−17.4)	—^§^	9.4 (4.6−14.3)	13.3 (8.5−18.2)	8.5^¶^ (4.5−12.5)	10.8 (6.8−14.7)	12.8 (8.9−16.8)
Idaho	8.7 (5.3−12.1)	12.9^¶^ (9.5−16.2)	8.4^¶^ (5.5−11.3)	11.4 (8.4−14.3)	11.4 (8.5−14.3)	—^§^	12.5 (3.7−21.3)	10.5 (1.7−19.3)	7.7^¶^ (3.8−11.7)	9.6 (5.7−13.6)	13.3 (9.3−17.2)
Illinois	8.0 (5.2−10.9)	11.3^¶^ (8.5−14.1)	7.5^¶^ (4.8−10.1)	9.0 (6.3−11.7)	11.5 (8.8−14.2)	6.1 (0.0−18.6)	14.1 (1.6−26.6)	9.4 (0.0−21.9)	9.6 (5.6−13.7)	7.9 (3.9−12.0)	10.9 (6.9−15.0)
Indiana	7.6 (4.9−10.3)	9.5^¶^ (6.8−12.2)	4.0^¶^ (1.4−6.6)	9.7 (7.1−12.3)	10.1 (7.5−12.7)	—^§^	9.6 (0.0−22.1)	8.9 (0.0−21.4)	4.4^¶^ (0.0−9.5)	7.2 (2.1−12.2)	11.5 (6.5−16.5)
Iowa	5.3 (2.7−7.9)	8.8^¶^ (6.2−11.3)	4.7^¶^ (2.2−7.1)	5.8^¶^ (3.3−8.2)	9.1 (6.7−11.6)	—^§^	11.7 (1.4−22.0)	6.9 (0.0−17.2)	6.2 (2.3−10.1)	5.9 (2.0−9.8)	8.1 (4.2−12.0)
Kansas	6.5 (4.3−8.7)	9.8^¶^ (7.5−12.0)	4.5^¶^ (2.3−6.8)	7.6^¶^ (5.3−9.8)	10.3 (8.0−12.5)	4.6 (0.0−10.4)	9.1 (3.4−14.9)	8.1 (2.4−13.9)	5.8^¶^ (2.5−9.1)	6.4^¶^ (3.2−9.7)	10.2 (6.9−13.4)
Kentucky	5.1 (2.4−7.8)	7.6^¶^ (4.9−10.3)	3.6^¶^ (0.9−6.4)	5.1^¶^ (2.4−7.8)	8.6 (5.8−11.3)	—^§^	—^§^	6.5 (0.0−16.3)	4.8 (0.2−9.4)	4.1^¶^ (0.0−8.7)	8.8 (4.2−13.3)
Louisiana	8.0 (5.4−10.7)	8.6 (5.9−11.2)	6.6 (4.1−9.1)	8.5 (6.0−11.0)	9.0 (6.5−11.5)	5.0 (0.0−13.9)	7.7 (0.0−16.6)	10.0 (1.1−18.9)	4.5^¶^ (0.0−9.0)	9.0 (4.4−13.6)	9.9 (5.3−14.5)
Maine	7.7 (4.7−10.8)	13.6^¶^ (10.5−16.6)	9.8 (7.1−12.5)	8.6^¶^ (5.9−11.3)	12.4 (9.7−15.0)	—^§^	—^§^	10.7 (0.0−21.5)	7.1^¶^ (3.4−10.8)	9.0^¶^ (5.3−12.7)	13.5 (9.8−17.2)
Maryland	6.9 (3.8−10.0)	10.9^¶^ (7.8−14.0)	5.8^¶^ (2.9−8.7)	8.0^¶^ (5.1−10.9)	11.3 (8.4−14.2)	5.4 (0.0−14.6)	11.2 (1.9−20.4)	9.6 (0.4−18.9)	5.9^¶^ (2.4−9.5)	6.8^¶^ (3.2−10.4)	11.0 (7.4−14.6)
Massachusetts	8.7 (5.7−11.8)	13.3^¶^ (10.2−16.3)	8.3^¶^ (5.3−11.3)	10.8 (7.8−13.8)	12.6 (9.6−15.6)	5.6 (0.0−13.7)	11.4 (3.3−19.5)	11.2 (3.1−19.3)	8.1^¶^ (4.0−12.1)	8.9 (4.9−13.0)	12.7 (8.7−16.8)
Michigan	5.6 (3.1−8.1)	9.8^¶^ (7.3−12.3)	4.4^¶^ (1.9−6.9)	6.8^¶^ (4.3−9.3)	9.7 (7.2−12.2)	5.5 (0.0−13.4)	7.9 (0.0−15.8)	8.0 (0.1−15.9)	5.5^¶^ (1.8−9.3)	5.7^¶^ (2.0−9.5)	9.9 (6.1−13.7)
Minnesota	6.1 (3.6−8.6)	10.2^¶^ (7.7−12.6)	5.6^¶^ (3.3−7.9)	7.7 (5.4−10.0)	9.7 (7.4−12.0)	4.3 (0.0−12.5)	12.3 (4.1−20.5)	8.1 (0.0−16.2)	5.6^¶^ (2.2−9.0)	5.9^¶^ (2.5−9.3)	10.0 (6.6−13.4)
Mississippi	5.3 (2.9−7.7)	7.0 (4.6−9.4)	5.8 (3.5−8.0)	4.6^¶^ (2.4−6.9)	7.7 (5.5−9.9)	3.1 (0.0−23.0)	—^§^	7.4 (0.0−27.3)	3.7^¶^ (0.0−8.6)	4.5^¶^ (0.0−9.3)	10.2 (5.4−15.1)
Missouri	6.0 (3.3−8.6)	9.4^¶^ (6.7−12.0)	5.8^¶^ (3.4−8.3)	6.6^¶^ (4.2−9.1)	9.4 (6.9−11.8)	5.2 (0.0−12.0)	5.1 (0.0−12.0)	8.0 (1.2−14.8)	3.9^¶^ (0.0−8.0)	6.8 (2.7−10.8)	9.8 (5.7−13.9)
Montana	5.6 (2.4−8.8)	11.0^¶^ (7.8−14.3)	5.6^¶^ (2.9−8.3)	7.5 (4.9−10.2)	9.8 (7.1−12.5)	—^§^	7.9 (1.1−14.6)	8.3 (1.5−15.0)	5.3^¶^ (1.4−9.1)	7.2 (3.3−11.0)	10.3 (6.5−14.2)
Nebraska	6.8 (4.4−9.1)	9.0 (6.7−11.4)	5.3^¶^ (2.9−7.6)	7.4 (5.0−9.7)	9.6 (7.2−11.9)	6.3 (0.0−14.5)	11.8 (3.5−20.0)	7.7 (0.0−16.0)	6.7 (2.7−10.6)	6.3 (2.3−10.3)	9.5 (5.5−13.5)
Nevada	9.8 (5.5−14.0)	13.3 (9.0−17.5)	14.3 (10.3−18.3)	9.2 (5.2−13.2)	12.4 (8.4−16.3)	—^§^	13.6 (6.2−21.0)	11.7 (4.3−19.1)	8.9^¶^ (3.4−14.3)	9.0^¶^ (3.5−14.4)	14.5 (9.0−19.9)
New Hampshire	8.5 (5.2−11.8)	13.1^¶^ (9.8−16.4)	5.5^¶^ (2.6−8.4)	11.2 (8.3−14.1)	12.4 (9.5−15.3)	—^§^	—^§^	10.9 (3.2−18.6)	5.9^¶^ (1.8−10.0)	9.6 (5.5−13.7)	12.3 (8.2−16.5)
New Jersey	6.2 (3.2−9.1)	10.3^¶^ (7.3−13.2)	5.4^¶^ (2.4−8.3)	6.8^¶^ (3.8−9.7)	10.6 (7.7−13.5)	4.8 (0.0−12.7)	8.8 (0.9−16.8)	8.4 (0.4−16.3)	6.8 (3.0−10.5)	5.9^¶^ (2.1−9.7)	10.1 (6.3−13.9)
New Mexico	9.1 (6.0−12.2)	11.5 (8.4−14.6)	9.1 (6.1−12.1)	9.0 (6.0−12.0)	11.9 (8.9−14.8)	—^§^	10.7 (2.9−18.4)	10.1 (2.3−17.8)	8.9 (4.0−13.8)	8.7 (3.8−13.6)	12.9 (8.0−17.8)
New York	7.7 (5.0−10.4)	11.4^¶^ (8.6−14.1)	6.9^¶^ (4.4−9.4)	9.5 (7.1−12.0)	10.9 (8.4−13.4)	7.0 (0.8−13.2)	9.8 (3.7−16.0)	10.2 (4.0−16.4)	7.1^¶^ (3.2−11.0)	7.9 (4.0−11.8)	11.7 (7.8−15.6)
North Carolina	7.3 (4.8−9.8)	8.9 (6.4−11.4)	4.4^¶^ (1.7−7.1)	7.6^¶^ (4.9−10.3)	10.3 (7.6−13.0)	3.9 (0.0−12.4)	9.7 (1.1−18.3)	8.8 (0.3−17.4)	5.3^¶^ (1.4−9.2)	6.6^¶^ (2.7−10.5)	10.5 (6.6−14.4)
North Dakota	6.4 (3.5−9.3)	9.5^¶^ (6.6−12.4)	5.6^¶^ (3.0−8.2)	7.4 (4.8−10.0)	9.6 (7.0−12.2)	—^§^	—^§^	7.7 (0.0−17.4)	5.0^¶^ (0.8−9.2)	6.5 (2.3−10.8)	9.3 (5.1−13.6)
Ohio	5.2 (2.6−7.8)	8.7^¶^ (6.1−11.3)	4.8^¶^ (2.4−7.1)	5.9^¶^ (3.6−8.2)	8.7 (6.4−11.0)	4.4 (0.0−12.2)	9.1 (1.3−16.9)	7.1 (0.0−14.9)	4.0^¶^ (0.0−8.3)	5.0^¶^ (0.7−9.2)	9.5 (5.3−13.8)
Oklahoma	5.4 (3.2−7.6)	6.8 (4.6−8.9)	3.0^¶^ (0.7−5.2)	5.9 (3.6−8.1)	7.7 (5.5−10.0)	2.3 (0.0−8.0)	5.5 (0.0−11.2)	6.5 (0.8−12.1)	3.2 (0.0−7.3)	5.2 (1.1−9.2)	8.3 (4.3−12.3)
Oregon	10.0 (6.5−13.5)	13.8^¶^ (10.3−17.3)	10.0^¶^ (6.9−13.0)	11.8 (8.7−14.9)	12.8 (9.7−15.9)	—^§^	13.6 (4.0−23.3)	12.0 (2.4−21.6)	8.3^¶^ (3.8−12.7)	10.6 (6.2−15.1)	14.1 (9.6−18.5)
Pennsylvania	6.9 (4.0−9.8)	9.8^¶^ (6.9−12.7)	3.7^¶^ (1.0−6.5)	8.7 (5.9−11.4)	10.1 (7.3−12.8)	6.6 (0.0−19.5)	—^§^	8.5 (0.0−21.4)	6.1 (1.7−10.5)	6.8 (2.4−11.2)	10.1 (5.7−14.5)
Rhode Island	8.2 (4.8−11.6)	11.2 (7.8−14.6)	9.6 (6.7−12.4)	8.3 (5.5−11.1)	10.9 (8.1−13.7)	—^§^	10.6 (0.2−20.9)	9.7 (0.0−20.0)	7.5^¶^ (3.1−11.8)	7.5^¶^ (3.2−11.9)	12.0 (7.6−16.3)
South Carolina	6.6 (4.2−9.1)	9.5^¶^ (7.1−12.0)	6.0^¶^ (3.6−8.3)	8.0 (5.7−10.4)	9.2 (6.9−11.6)	3.1 (0.0−11.2)	20.5^¶^ (12.4−28.6)	9.0 (0.9−17.1)	5.7^¶^ (1.9−9.5)	6.1^¶^ (2.3−9.9)	11.0 (7.2−14.8)
South Dakota	4.5 (2.0−6.9)	7.5^¶^ (5.0−10.0)	2.2^¶^ (0.0−4.6)	5.4^¶^ (3.0−7.9)	7.9 (5.5−10.3)	—^§^	—^§^	6.3 (0.6−11.9)	3.6^¶^ (0.0−7.2)	4.9 (1.3−8.5)	7.4 (3.8−11.0)
Tennessee	9.9 (6.8−12.9)	9.4 (6.4−12.4)	9.3 (6.5−12.1)	9.2 (6.4−12.0)	10.2 (7.4−13.0)	—^§^	9.7 (0.0−25.6)	10.1 (0.0−26.0)	7.6^¶^ (3.0−12.3)	7.6^¶^ (3.0−12.3)	12.7 (8.1−17.4)
Texas	9.7 (6.6−12.7)	12.0 (9.0−15.1)	8.0^¶^ (4.9−11.1)	10.6 (7.5−13.7)	12.7 (9.6−15.9)	7.2 (0.0−15.6)	11.7 (3.2−20.1)	11.2 (2.8−19.6)	8.8 (4.1−13.5)	10.3 (5.6−14.9)	12.4 (7.7−17.1)
Utah	7.8 (5.1−10.6)	11.0^¶^ (8.3−13.8)	6.6^¶^ (3.8−9.4)	9.4 (6.6−12.2)	11.6 (8.8−14.4)	—^§^	11.3 (0.6−22.1)	9.3 (0.0−20.0)	7.5 (3.3−11.7)	8.4 (4.2−12.6)	10.6 (6.4−14.9)
Vermont	7.7 (4.3−11.1)	14.4^¶^ (11.0−17.8)	8.4^¶^ (5.4−11.4)	9.8^¶^ (6.8−12.7)	12.9 (10.0−15.9)	—^§^	—^§^	11.1 (2.6−19.6)	6.5^¶^ (2.4−10.5)	7.7^¶^ (3.7−11.8)	14.6 (10.5−18.6)
Virginia	6.0 (3.5−8.6)	9.2^¶^ (6.6−11.8)	5.2^¶^ (2.6−7.8)	6.7^¶^ (4.1−9.3)	9.7 (7.0−12.3)	4.0 (0.0−13.1)	9.1 (0.1−18.2)	8.4 (0.0−17.4)	4.5^¶^ (0.7−8.2)	5.8 (2.0−9.6)	9.5 (5.7−13.3)
Washington	8.1 (5.1−11.0)	13.8^¶^ (10.8−16.8)	7.1^¶^ (4.3−9.9)	10.7 (7.9−13.5)	13.0 (10.2−15.8)	9.8 (3.1−16.5)	10.8 (4.1−17.5)	11.0 (4.3−17.7)	8.9^¶^ (5.3−12.5)	8.7^¶^ (5.1−12.3)	12.8 (9.2−16.4)
West Virginia	4.7 (2.4−6.9)	7.0^¶^ (4.8−9.2)	3.5^¶^ (1.3−5.7)	4.7^¶^ (2.4−6.9)	7.5 (5.3−9.7)	—^§^	—^§^	6.0 (0.0−15.0)	3.5^¶^ (0.0−7.2)	4.9 (1.2−8.6)	8.2 (4.6−11.9)
Wisconsin	6.2 (3.5−8.9)	9.4^¶^ (6.7−12.1)	5.7^¶^ (3.1−8.2)	7.2 (4.7−9.8)	9.1 (6.6−11.7)	—^§^	7.9 (0.0−20.3)	7.7 (0.0−20.1)	5.0^¶^ (0.9−9.0)	6.8 (2.7−10.9)	9.2 (5.2−13.3)
Wyoming	8.4 (5.3−11.5)	9.9 (6.8−13.0)	7.8^¶^ (4.9−10.7)	7.6^¶^ (4.7−10.5)	11.0 (8.1−13.9)	—^§^	11.0 (2.8−19.3)	9.0 (0.7−17.3)	6.1^¶^ (1.7−10.5)	6.5 (2.1−10.9)	11.7 (7.4−16.1)

**Abbreviations:** CI = confidence interval; Ref = referent group.

* Weighted percentages are presented.

^†^ Blacks and whites are non-Hispanic; Hispanic persons could be of any race. Other racial/ethnic group not reported because of small sample sizes but included in overall estimates and estimates by other demographic characteristics.

^§^ Data where the sample sizes were <50 were considered unstable and were not reported.

^¶^ p<0.05 for t-test comparing differences by demographic groups to the referent group.

## Discussion

Overall, the proportion of adults meeting fruit and vegetable intake recommendations remained low in 2015, with more persons meeting recommendations for fruit intake than vegetable intake, and with substantial variations by state, age, sex, race/ethnicity, and IPR. Consistent with earlier studies of BRFSS data (*5*,*6*), a higher percentage of women than men and a higher percentage of adults aged ≥51 years than persons aged 18–30 years met recommendations for fruit and vegetable intake. Findings are also consistent with earlier work demonstrating larger IPR-related disparities in vegetable than fruit intake as well as a significantly higher prevalence of meeting recommendations for fruit intake among blacks and Hispanics than among whites (*5*). However, this analysis did not observe previously reported significantly lower prevalences of meeting recommendations for vegetable intake among Hispanics and blacks compared with whites; that study measured the percentage of respondents who consumed vegetables more than three times per day rather than the age- and sex-specific cup-equivalent measure used in the current analysis.

Because fruit and vegetable consumption affects multiple health outcomes, including cardiovascular disease, type 2 diabetes, some cancers, and obesity (*1*) and is currently low among adults in all states and demographic subgroups, continued efforts are needed to identify and address barriers to fruit and vegetable consumption. A recent review identified several barriers, including high cost, limited availability and access, and perceived lack of preparation time (*7*,*8*). The CDC Guide to Strategies to Increase the Consumption of Fruits and Vegetables^¶^ identifies 10 strategies to increase access to and improve the availability of fruits and vegetables. Examples include starting or expanding farm-to-institution programs in childcare, schools, hospitals, workplaces, and other institutions; improving access to retail stores and markets that sell high quality fruits and vegetables; and ensuring access to fruits and vegetables in cafeterias and other food service venues in worksites, hospitals, and universities. To address cost, the U.S. Department of Agriculture Food Insecurity Nutrition Incentive (FINI) grant program** supports projects to increase the purchase of fruits and vegetables among low-income consumers participating in the Supplemental Nutrition Assistance Program, by providing incentives at the point of purchase; FINI projects are currently underway in 26 states.^††^

The findings in this report are subject to at least six limitations. First, estimates did not include non-100% fruit juice or fried potatoes because the BRFSS questionnaire instructs respondents to exclude them. These foods were excluded from BRFSS because federal dietary guidelines recommend limiting foods and beverages with added sugars and solid fats such as these (*1*); estimates therefore represent intake from healthier sources. Including these additional sources of fruits and vegetables results in 4%–6% higher estimates for fruit intake and 30%–44% higher estimates for vegetable intake (*4*). Second, because the data are self-reported, they are subject to biases that might result in either overestimates or underestimates of actual fruit and vegetable consumption, and different demographic groups might differentially misreport intake.^§§^ Third, the BRFSS survey excludes persons living in nursing homes, long-term care facilities, military installations, and correctional institutions, and thus these data are not generalizable to the entire U.S. population. Moreover, territories were excluded from this analysis because the scoring algorithms were derived from NHANES, which excludes territories. Fourth, using the scoring algorithms to estimate intake might have resulted in measurement error. However, previous analyses showed that applying prediction equations to 2011 BRFSS frequency data yielded estimates comparable to 2007–2010 national estimates that used more accurate 24-hour recalls (*4*). Fifth, approximately 13% of participants had fruit and vegetable data missing (58,949 participants). These respondents included a higher proportion of older adults and persons with IPR <1.25, similar to a previous study (*4*). A sensitivity analysis demonstrated that estimates did not change when persons with complete data for fruit intake, but not vegetables, or when persons with complete data for vegetable intake but not fruit were included. Finally, among the 375,306 eligible participants who had complete information for fruit and vegetable intakes and resided in the study area, 13% (55,891 participants) were excluded because they did not report household income. Estimated percentages of persons meeting recommendations were similar when income was imputed for persons with missing household income based on age, sex, and race/ethnicity. The estimates without imputation are presented to be consistent with previous studies (*2*,*4*) which allow states to compare estimates for surveillance purposes.

Despite the positive health benefits of consuming fruits and vegetables, the findings from this study corroborate data showing that the vast majority of adults consume insufficient amounts, with lower intakes among men, young adults, and adults living in poverty. For most states, the only source of state-level nutritional data for adults is fruit and vegetable intake data from BRFSS. States can use this information to inform the development of policies and programs that help all adults regardless of sociodemographic group to consume more fruits and vegetables and thus help prevent costly chronic diseases.

**Summary**

**What is already known about this topic?**

Consuming enough fruits and vegetables as part of an overall healthy diet reduces the risk of many chronic diseases, including cardiovascular disease, type 2 diabetes, some cancers, and obesity. However, the percentage of the adult population meeting fruit and vegetable intake recommendation is low. In 2013, 13.1% of respondents met fruit intake recommendations and 8.9% met vegetable recommendations.

**What is added by this report?**

Recent data show adults continue to consume too few fruits and vegetables; overall, 12.2% met fruit intake recommendations and 9.3% met vegetable intake recommendations during 2015. Consumption was lower among men, young adults, and adults with greater poverty, and varied by state. Among subgroups, the largest disparities in meeting the recommendation for fruit intake was by sex (15.1% among women compared with 9.2% among men), while the largest disparities in meeting the recommendation for vegetable intake was by poverty (11.4% among adults in the highest household income category compared with 7.0% among adults below or close to the poverty level).

**What are the implications for public health practice?**

States can use this information to inform the development of policies and programs that help all adults regardless of sociodemographic groups to consume more fruits and vegetables and thus help to prevent costly chronic diseases.

## Footnotes

* Appropriate for adults who engage in <30 minutes of moderate physical activity; more active adults might be able to consume more while staying within calorie needs. https://www.choosemyplate.gov/fruit; https://www.choosemyplate.gov/vegetables.

†
https://aspe.hhs.gov/2015-poverty-guidelines .

§ https://www.cdc.gov/brfss/annual_data/2015/2015_responserates.html.

¶
https://www.cdc.gov/obesity/downloads/fandv_2011_web_tag508.pdf .

**
https://nifa.usda.gov/program/food-insecurity-nutrition-incentive-fini-grant-program .

††
https://nifa.usda.gov/program/food-insecurity-nutrition-incentive-fini-grant-program .

§§ https://dietassessmentprimer.cancer.gov/concepts/.
